# The CI MuMuFe – A New MMN Paradigm for Measuring Music Discrimination in Electric Hearing

**DOI:** 10.3389/fnins.2020.00002

**Published:** 2020-01-23

**Authors:** Bjørn Petersen, Anne Sofie Friis Andersen, Niels Trusbak Haumann, Andreas Højlund, Martin J. Dietz, Franck Michel, Søren Kamaric Riis, Elvira Brattico, Peter Vuust

**Affiliations:** ^1^Center for Music in the Brain, Department of Clinical Medicine, Aarhus University and The Royal Academy of Music, Aarhus/Aalborg, Aarhus, Denmark; ^2^Center of Functionally Integrative Neuroscience, Aarhus University, Aarhus, Denmark; ^3^Audiological Clinic, Department of Otorhinolaryngology, Head and Neck Surgery, Aarhus University Hospital, Aarhus, Denmark; ^4^Oticon Medical, Smørum, Denmark

**Keywords:** cochlear implants, mismatch negativity, auditory discrimination, music perception, multi-feature paradigm

## Abstract

Cochlear implants (CIs) allow good perception of speech while music listening is unsatisfactory, leading to reduced music enjoyment. Hence, a number of ongoing efforts aim to improve music perception with a CI. Regardless of the nature of these efforts, effect measurements must be valid and reliable. While auditory skills are typically examined by behavioral methods, recording of the mismatch negativity (MMN) response, using electroencephalography (EEG), has recently been applied successfully as a supplementary objective measure. Eleven adult CI users and 14 normally hearing (NH) controls took part in the present study. To measure their detailed discrimination of fundamental features of music we applied a new multifeature MMN-paradigm which presented four music deviants at four levels of magnitude, incorporating a novel “no-standard” approach to be tested with CI users for the first time. A supplementary test measured behavioral discrimination of the same deviants and levels. The MMN-paradigm elicited significant MMN responses to all levels of deviants in both groups. Furthermore, the CI-users’ MMN amplitudes and latencies were not significantly different from those of NH controls. Both groups showed MMN strength that was in overall alignment with the deviation magnitude. In CI users, however, discrimination of pitch levels remained undifferentiated. On average, CI users’ behavioral performance was significantly below that of the NH group, mainly due to poor pitch discrimination. Although no significant effects were found, CI users’ behavioral results tended to be in accordance with deviation magnitude, most prominently manifested in discrimination of the rhythm deviant. In summary, the study indicates that CI users may be able to discriminate subtle changes in basic musical features both in terms of automatic neural responses and of attended behavioral detection. Despite high complexity, the new CI MuMuFe paradigm and the “no-standard” approach provided reliable results, suggesting that it may serve as a relevant tool in future CI research. For clinical use, future studies should investigate the possibility of applying the paradigm with the purpose of assessing discrimination skills not only at the group level but also at the individual level.

## Introduction

The cochlear implant (CI) represents a major breakthrough in the history of medicine and has meant a tremendous difference in the lives of thousands of people. After receiving this auditory prosthesis, patients with moderate to profound hearing loss are able to gain or regain the sense of hearing, allowing not only postlingually deafened adults to reestablish speech comprehension but also children with profound congenital hearing loss to acquire spoken language (Limb and Rubinstein, 2012). Moreover, recent technological refinements and a general rise in bilateral implantation have further improved implant outcomes over the last decades. Correspondingly, there has been a dramatic rise in the number of CI surgeries, and today >500,000 CI recipients worldwide use the device in their daily communication (source: EuroCIU).

Despite the success of CIs, some problems remain unsolved. Lack of temporal fine-structure, low spectral resolution, and a limited dynamic range in the CI signal are the cause of poor perception of pitch, timbre, and intensity (Drennan and Rubinstein, 2008). As a consequence, CI users experience challenges with perception of prosody (Peng et al., 2008) and emotional prosody (Hopyan-Misakyan et al., 2009; Nakata et al., 2012). Due to the complex temporal and tonal features of music, music listening is particularly challenging. This is indicated by reduced levels of music enjoyment (Gfeller et al., 2000; Lassaletta et al., 2008; Looi and She, 2010; Moran et al., 2016; Dritsakis et al., 2017a), poor perception of pitch (Gfeller et al., 2007; Zeng et al., 2014), impaired recognition of melodic contour (Galvin et al., 2007) and difficulties in identifying musical instruments (Driscoll, 2012; Kim et al., 2015; for a review see Jiam, 2017). Since improved perception of music represents a strong desire and could improve quality of life in CI users (Drennan and Rubinstein, 2008; Dritsakis et al., 2017b), a number of ongoing efforts aim to improve music perception with a CI (Petersen et al., 2012, 2014; Gfeller et al., 2015; Bedoin et al., 2018; Fuller et al., 2018; Jiam et al., 2019). Regardless of whether these efforts are of a rehabilitative or technological nature, it is imperative that measurements of the effect are valid and reliable.

In clinical context as well as in research, CI-users’ auditory perception skills are typically measured by behavioral methods. In recent years, however, electroencephalographic (EEG) methods have been successfully applied as a supplementary measure. EEG offers the opportunity to investigate auditory function with a high temporal resolution by recording event-related brain potentials (ERPs). Especially one ERP, the mismatch negativity (MMN) response, has proven to be a reliable and objective marker for CI users’ ability to accurately discriminate auditory stimuli (for a review see Näätänen et al., 2017). The MMN is a neural response elicited when a sensory input does not match the predicted pattern. Thus, the MMN indexes an error in the predictive coding of the environment, e.g., when a deviation in pitch, timbre, or rhythm occurs in a regular pattern of standard stimuli. The MMN is characterized by a greater negativity and it usually peaks 100–250 ms after deviation onset (Näätänen et al., 2001). Moreover, the MMN is an automatic response which means that it can be studied independently of the participant’s attention (Näätänen et al., 1978; Alho, 1992; Paavilainen et al., 1993). As such, recording of the MMN is particularly relevant in small children who are unable to provide subjective responses.

In normally hearing (NH) individuals, the amplitude and the latency of the MMN response are in general related to the deviation magnitude, such that large deviations yield larger MMN amplitudes with shorter latencies and vice versa (Kujala, 2007; Vuust et al., 2011). In CI users, MMN responses typically show trends of smaller amplitudes and longer latencies compared to NH controls (Titterington et al., 2003; Kelly, 2005; Roman, 2005; Timm et al., 2014). It should be emphasized, however, that some studies have been unable to demonstrate reliable MMN responses in CI users which may be attributed to reduced recruitment of the auditory cortex as a consequence of prelingual hearing loss and/or long duration of deafness (Zhang, 2011; Petersen et al., 2013; Näätänen et al., 2017).

Historically, the MMN has been recorded with oddball paradigms in which an occasional deviant is randomly introduced into sequences of standards (Näätänen, 1992), typically at a ratio of 2:8. Recently, multi-feature paradigms have been introduced in which the standards alternate with several types of deviants, thus allowing for recording of MMNs to several features. An early version of a multi-feature paradigm, “Optimum 1” (Näätänen et al., 2004; Pakarinen et al., 2007), was first used with adult CI-listeners by Sandmann et al. (2010). In their configuration, identical synthesized clarinet tones alternated with deviants in either pitch, intensity, or duration at one of four levels of deviation magnitude. The authors found that CI users produced smaller MMN amplitudes for frequency and intensity deviations compared to NH listeners and failed to show any magnitude-of-deviance effect. According to the authors, these difficulties in discriminating small changes in the acoustic properties of musical sounds could to some extent account for CI users’ poor perception and enjoyment of music.

With the purpose of creating a more complex and musically rich stimulation Vuust et al. (2011) introduced the “Musical Multi-feature” (MuMuFe) paradigm which instead of repeating notes presents arpeggiated triads in alternating keys. In two previous studies, we successfully adapted a version of the MuMuFe paradigm to investigate music discrimination skills in postlingually deaf adult and prelingually deaf adolescent CI users (Petersen et al., 2014; Timm et al., 2014). Both studies showed robust MMN responses to deviations in timbre and intensity in CI users. However, while the adult group failed to show robust MMN responses to rhythm, the adolescents failed to show robust MMN responses to pitch. Except for two magnitudes of the pitch deviant, these paradigms only contained one level of deviation which excluded assessment of discrimination thresholds. Recently, Hahne et al. (2016) carried out two experiments using modified versions of the MuMuFe, each presenting a different level of deviation magnitude. The authors reported marked effects of deviation magnitude on MMN amplitude across CI and NH groups. Furthermore, they found strong between-group differences attributed to CI users’ lower MMN responses to intensity, rhythm, and pitch. Interestingly, while postlingually deaf participants showed larger MMN responses than prelingually deaf participants, only the pitch condition showed a significant between-group difference.

The present study aimed to investigate CI users’ discrimination accuracy for changes in salient musical features at a high level of detail. For this purpose, we adapted a version of the MuMuFe MMN paradigm which presented four deviants at four levels of magnitude. Furthermore, to reduce recording time and at the same time increase the speed with which deviants are presented, we applied a “no-standard” approach (Pakarinen et al., 2010) to be tested with CI users for the first time. Unlike the original version of the paradigm in which every other pattern included a “standard” note, every pattern in the no-standard version presents one type of deviant, thus omitting the standards (Kliuchko et al., 2016). As a complementary measure, we applied a behavioral test which examined attentive discrimination of the same features and levels also presented in the MMN-paradigm.

We hypothesized that despite a high complexity, the MMN-paradigm would elicit significant MMN responses in CI users as well as NH controls. Furthermore, by presenting deviants at different levels, we hypothesized that MMN-amplitudes and behavioral measures would reflect deviation magnitude and thus, by extension, reflect the paradigm’s potential to estimate the resolution threshold at which CI-users are able to accurately discriminate different musical sounds. For potential validation of the paradigm’s viability in CI-research and possible revision, a key aim was to test whether an effect of level on the MMN strength was present for each feature in each group. Extending previous findings, we expected that CI-users’ overall MMN-responses would be significantly smaller in amplitude and longer in latency than those of NH controls.

## Materials and Methods

### Participants

Eleven experienced CI users (*M*^age^: 56.1 years; range 34–77 years; nine women) were recruited for the study through the Danish CI users’ organization and their online platform. The CI users had an average duration of deafness prior to CI of 24 years (range 0.5–56 years) and their mean experience with the CI was 7 years (range 1–14 years) Two CI users were bilaterally implanted and four used a hearing aid on the side contralateral to their CI. Nine participants reported ability to speak on the phone, indicating a high level of CI outcome (see Table 1 for details).

**TABLE 1:** Demographic and clinical characteristics of the 11 CI users.

**Group ID**	**Age at project**	**Duration of deafness**	**CI experience**	**Number of**	**Side of implant**	**Hearing aid**	**Telephone ability**
	**start (years)**	**prior to CI (years)**	**(years)**	**implants**	**used for tests**		
CI01	65–70	32	7	1	L	x	x
CI02	45–50	36	10	1	L	–	x
CI03	50–55	20	5	1	R	–	x
CI04	55–60	17	9	2	R	–	x
CI05	60–65	56	4	1	L	–	x
CI06	60–65	10	14	2	L	–	x
CI07	45–50	17	7	1	R	x	x
CI08	65–70	55	8	1	R	–	–
CI09	75–80	8	4	1	L	–	–
CI10	35–40	9	1	1	R	x	x
CI11	35–40	0	8	1	L	x	x
Mean	56	24	7				

*Hearing aid: the participant wears a hearing aid on the ear contralateral to the implanted ear. Telephone ability: the participant reports ability to communicate via telephone via CI or CI + HA.*

Fourteen older adults with normal hearing (*M*^age^: 63.4 years; range 56–77 years; seven women) were included for reference and validation of the paradigm, recruited via social media. Comparison of age by means of a *t*-test showed that the mean age did not differ significantly between the two groups (*p* = 0.079). Normality of hearing was assured by passing of an online hearing test which adaptively estimated a threshold for perception of words and numbers in background noise^1^.

All participants in both groups met criteria for being non-musicians, i.e., <5 years of formal singing or instrument training and no or only moderate formal knowledge of music. All participants received oral and written information about the study before giving consent to participate. The study was conducted in accordance with the Helsinki declaration and approved by the Research Ethics Committee of the Central Denmark Region. No monetary compensation was provided.

The study is part of a broader project which also investigates the neural plasticity underlying adaptation to the CI in naive implantees and the potential beneficial effects of a novel sound compression strategy in the Oticon Medical Neuro CI system (VoiceGuard) on the music perception of CI users. In addition to the MuMuFe, the participants were also presented with a free-listening EEG-paradigm, presenting real music to be reported in a separate paper.

### Stimuli

The CI MuMuFe MMN-paradigm is adapted from the musical multifeature paradigm developed by Vuust et al. (2011) and integrates the no-standards approach from Kliuchko et al. (2016). Four different deviants, representing basic parameters of music, are embedded in an Alberti bass configuration, a four-tone arpeggiated accompaniment pattern, typically used in classical music. Deviants are presented randomly at four levels of magnitude: small (S), medium (M), large (L), and extra-large (XL), adding to a total of 16 variants. In all cases, the deviants occur at the place of the third note in the pattern.

**FIGURE 1:**
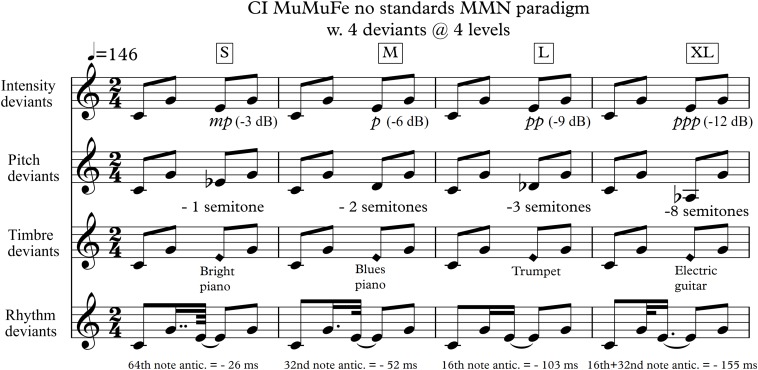
The CI MuMuFe no standards 4 deviants/4 levels MMN paradigm. The paradigm is randomly presented in four keys: C, Eb, Gb, and A major. Lowest note is Ab3 (208 Hz), highest note is E5 (659 Hz). S, small; M, medium; L, large; XL, extra-large.

The paradigm incorporates the following deviants and levels: (1) An intensity deviant created by decreasing the intensity of the regular note with 3, 6, 9, or 12 decibel (dB). (2) A pitch deviant created by lowering the regular note with either one, two, three, or eight semitones. (3) A timbre deviant created by exchanging the regular piano sound with either a bright piano sound, a blues piano sound, a trumpet sound, or a guitar sound. (4) A rhythm deviant created by shortening the second note by 26, 52, 103, or 155 ms while at the same time lengthening the third note accordingly to avoid a silent gap. The four displacements of the third note equivalates a 64th-, a 32nd-, a 16th-, and a dotted 16th-note, respectively, at a tempo of 146 BPM (Figure 1).

Piano sounds were created using the virtual piano Alicia’s Keys (Native Instruments). The four deviant sounds were taken from the sample library of the software sampler Halion SE in Cubase Pro 8 (version 8.0.30). The sounds were exported in mono with a sampling frequency of 44.100 Hz and subsequently modified with an 18 ms rise and fall and amplitude normalized in Adobe Audition (2015.0 Release). Modification of the intensity and rhythm deviants was performed similarly.

Each tone was 200 ms long and was presented with an interstimulus interval (ISI) of 5 ms. Following three repetitions of each deviant, a change of key occurred. Notes were kept in the middle register of the piano going from Ab3 (208) to E5 (659 Hz). The order of the four possible keys (C, Eb, Gb, and A) and of deviants was pseudorandomized using Matlab (R2016a).

The paradigm was presented using the Presentation software (Neurobehavioral systems) and presented each deviant level 96 times incorporating a total of 6144 (4 ^∗^ 4 ^∗^ 4 ^∗^ 96) stimuli. The stimuli were presented in four blocks lasting 8 min with a pause of approximately 1 min between blocks, adding to a total recording time of approximately 35 min.

### Procedure

Electroencephalography was recorded at the MINDLab EEG facility of Danish Neuroscience Center, Aarhus, Denmark, using a BrainAmp amplifier system (Brain Products, Gilching, Germany) with a 32-electrode cap with electrodes placed according to the international 10/20 system. Electrode numbers 28 and 32 were placed beside and over the left eye to record the electrooculogram (EOG). For CI users, some parietal channels could not be used because of interference with the CI transmitter coil. Data were recorded with a sampling rate of 1000 Hz using the position FCz as reference. Electrode impedances were maintained <25 kΩ prior to data acquisition.

During EEG recordings participants sat in an electrically and acoustically shielded room and were instructed to ignore the auditory stimuli and focus on a movie in which the audio was muted. For all participants, the sound level was individually adjusted to a comfortable level from a defined starting point of 65 dB SPL. NH participants received sound bilaterally through in-ear Shure headphones. To ensure comparable test conditions, CI users received sound monaurally. Bilateral CI users were asked to use their preferred implant; bimodally aided participants were asked to remove their hearing aid. To rule out any residual hearing, CI users received the stimuli directly in their implant via audio cable with microphones muted. CI users used their everyday processor settings during the EEG session. In cases in which the CI speech processor lacked a direct audio input, a spare processor was programmed with the participant’s settings and used instead.

### EEG Data Analysis and Statistics

The EEG data were preprocessed with the FieldTrip Toolbox for Matlab (Oostenveld et al., 2011). The data were first downsampled to 250 Hz and bandpass filtered between 1 and 25 Hz. Unused channels and other bad channels were replaced by interpolation of neighboring channels for CI users (mean: 2.5; range: 1–3 electrodes) and for NH controls (mean: 0.1; range: 0–1 electrode). This was achieved with the FieldTrip *ft_channelrepair* function applying the default interpolation based on an average weighted by the distance of neighboring channels. Eye movement and CI artifact components were isolated with the infomax independent component analysis (ICA) algorithm for EEG (Makeig et al., 1996; Delorme et al., 2007). A clear vertical eye movement component was visually observed and removed in all CI users and all except one of the NH controls. A salient horizontal eye movement component was visually identified and removed in 9 of 11 CI users and 10 of the 14 NH. For each CI user, one to eight CI artifact components were visually identified and removed, based on whether their topographical centroids were located above the implant site (Viola et al., 2011; Näätänen et al., 2017) and their waveforms were distinguishable from ordinary auditory evoked responses and neurophysiological oscillations.

Following the approach of previous MMN studies with CI-users in which consistent mastoid signals could not be obtained (Bishop and Hardiman, 2010; Sandmann et al., 2010; Zhang, 2011) data were re-referenced to the mean across all channels. Subsequently, trials were extracted using a 100 ms baseline corrected pre-stimulus window and a 400 ms post-stimulus time window. An exception was the responses to the rhythm deviants, where the baseline was corrected from −100 to 0 ms in relation to the onset of the preceding note (i.e., the second note). We implemented this measure in order to avoid the possibility that the temporal variance would affect the baseline. Any undetected noisy trials with amplitudes exceeding ±100 μV were automatically detected and removed (0.2% of all trials). As in the previous no-standard MuMuFe studies (Haumann et al., 2016; Kliuchko et al., 2016; Bonetti et al., 2017, 2018), the ERPs to notes 1, 2, and 4 were applied as the best option for standards (see Supplementary Material). The trials were averaged, and the standard responses subtracted from the deviant responses to identify potential MMN responses.

Statistical analysis on the MMN difference waves was conducted by following the clinical conventions of obtaining the average Fz electrode amplitude (Duncan et al., 2009; Näätänen et al., 2017) across a 30 ms time window centered on the feature-specific negative peak in the grand average ERP waveform (Näätänen et al., 2017). With the current stimulus paradigm and population samples, similar but slightly differing feature-specific MMN peak latencies were measured for each feature across groups: 148 ms for intensity, 156 ms for pitch, 132 ms for rhythm, and 152 ms for timbre MMN. For latency analysis, individual participants’ peaks were identified as the most negative peak in the difference wave between 100 and 250 ms.

The statistical results for the MMN amplitudes and latencies were obtained using a three-way mixed effects ANOVA model using the IBM SPSS v25 software package. The tested between-subject factor was Group (NH controls, CI users) and within-subject factors were deviant Feature (Intensity, Pitch, Timbre, Rhythm) and Level (S, M, L, XL). Since Mauchly’s test of sphericity showed violation of the sphericity assumption for the Feature factor (*p* = 0.004) with respect to MMN amplitude, the Greenhouse–Geisser correction for non-sphericity was applied for the Feature factor. According to our *a priori* hypotheses, we were interested in the potential main effect of Group and Level, the potential interaction between Group and Feature, as well as the three-way interaction between Group, Feature, and Level. We therefore focused our statistical analyses on these four terms, and thus adjusted the alpha level for the ANOVA by a factor of four to account for the four terms tested (α = 0.05/4 = 0.0125).

Furthermore, to first test for significant MMN responses for all types of deviants, we conducted one-sample *t*-tests for the MMN amplitudes against 0 μV for each Level and Feature in each Group, applying Bonferroni-correction of the alpha-level [α = 0.05/(4^∗^4) = 0.0031]. In order to fully investigate the discrimination resolution of the paradigm, we carried out planned comparisons of the Level factor for each Feature and each Group on the MMN amplitudes, i.e., six Level contrasts (3 + 2 + 1) for each of the four Features, using paired-samples *t*-tests with Bonferroni-correction of the significance level for multiple comparisons [α = 0.05/(6^∗^4) = 0.0021].

### Behavioral Test

In addition to the EEG measurements, all participants completed a three-alternative forced choice (3-AFC) test to obtain a behavioral measurement of the same music parameters and levels of magnitude as presented in the MMN paradigm.

A four-tone musical pattern, like the one presented in the MMN paradigm, was presented twice in the standard and once in the deviant condition [*p*(deviant) = 0.33]. The participants were hereafter instructed to manually choose the deviant pattern based on a pictorial representation on the computer screen. The deviant could occur in either the first, second, or third pattern in a randomized order. Each of the 16 deviants were presented six times adding up to a total of 96 trials. The scores were converted to percent correct hit rates for each deviant condition.

The 3-AFC data did not meet the criteria for normal distribution (NH: Shapiro–Wilk, *p* = 0.10^–6^–0.131; CI: Shapiro–Wilk, *p* = 0.10^–7^–0.535). Consequently, to test whether hit rates differed significantly from chance level, one-sample Wilcoxon signed rank-tests against the value 33.3% were conducted. Again, we focused our statistical analyses on the potential main effects of Group and Level, as well as the potential interactions between Group and Feature and Group, Feature and Level. NH and CI hit rates were compared with a Mann–Whitney *U*-test, and effects of Level (S, M, L, XL) on hit rates were tested with Friedman’s ANOVA, both with a Bonferroni-corrected alpha level of factor two (α = 0.05/2 = 0.025). Given that the standard Friedman’s ANOVA does not include tests of interaction effects, we report Bonferroni-corrected comparisons for the potential Group by Feature interaction (α = 0.05/4 = 0.0125) together with the other *post hoc* comparisons. Finally, the potential three-way interaction was implicitly tested as part of our planned comparisons of the Level factor for each feature and each group which was tested with Wilcoxon signed rank tests with Bonferroni-corrected significance levels [α = 0.05/(6 ^∗^ 4) = 0.0021].

### Correlation Analysis

To identify possible predictive factors and relationships, we performed a multiple regression analysis of the CI users’ MMN amplitudes, behavioral hit rates, clinical data, and music appreciation data. Clinical factors were age, age at hearing loss, age at implantation, duration of hearing loss prior to CI, and duration of CI experience. Music appreciation data included music listening habits (hours/week), level of music enjoyment (1–7), and rating of quality of music with CI (mean VAS score across seven bipolar adjective descriptors). The latter were extracted from responses given in a revised version of the IOWA musical background questionnaire on the online platform SurveyXact.

## Results

### Overall MMN Responses

All deviant types and levels elicited statistically significant MMN responses in both the NH group (*p* < 10^–4^) and in the CI group (*p* < 0.001) (Table 2 and Supplementary Figure 1). For both groups, the Bonferroni-corrected alpha level of α = 0.05/(4 ^∗^ 4) = 0.0031 was used.

**TABLE 2:** MMN amplitudes.

**Feature**	**Level**	**NH Controls**				**CI users**			
		**Latency in ms (SD)**	**Amplitude in μV (SD)**	***t***	***P***	**Latency in ms (SD)**	**Amplitude in μV (SD)**	***t***	***p***
Intensity	S	161 (50)	−0.63 (0.34)	−6.94	<10^–4^**	151 (24)	−0.87 (0.57)	−5.12	<0.001**
	M	175 (47)	−0.72 (0.44)	−6.00	<10^–4^**	164 (53)	−0.84 (0.53)	−5.31	<0.001**
	L	169 (39)	−0.89 (0.55)	−6.03	<10^–4^**	162 (35)	−1.03 (0.54)	−6.35	<10^–4^**
	XL	173 (32)	−1.11 (0.49)	−9.25	<10^–6^**	150 (30)	−1.38 (0.70)	−6.54	<10^–4^**
Pitch	S	154 (32)	−1.33 (0.69)	−7.16	<10^–4^**	181 (32)	−0.98 (0.46)	−7.12	<10^–4^**
	M	171 (41)	−1.12 (0.65)	−6.48	<10^–4^**	176 (40)	−0.90 (0.31)	−9.64	<10^–5^**
	L	182 (47)	−1.18 (0.72)	−6.10	<10^–4^**	173 (32)	−0.91 (0.69)	−4.39	0.001**
	XL	151 (17)	−1.83 (0.87)	−7.90	<10^–5^**	174 (36)	−0.92 (0.47)	−6.51	<10^–4^**
Timbre	S	158 (41)	−1.04 (0.54)	−7.21	<10^–4^**	148 (12)	−1.43 (0.35)	−13.67	<10^–7^**
	M	174 (40)	−0.82 (0.47)	−6.49	<10^–4^**	192 (40)	−0.50 (0.41)	−4.05	0.002**
	L	175 (39)	−1.46 (0.70)	−7.74	<10^–5^**	165 (28)	−1.24 (0.60)	−6.88	<10^–4^**
	XL	145 (31)	−1.47 (0.72)	−7.61	<10^–5^**	157 (36)	−1.57 (0.78)	−6.72	<10^–4^**
Rhythm	S	161 (42)	−0.84 (0.36)	−8.59	<10^–5^**	203 (30)	−0.63 (0.53)	−3.93	0.003**
	M	143 (32)	−1.34 (0.64)	−7.76	<10^–5^**	164 (41)	−0.84 (0.54)	−5.10	<0.001**
	L	150 (39)	−1.55 (0.81)	−7.13	<10^–5^**	166 (46)	−1.48 (0.82)	−5.99	<0.001**
	XL	149 (48)	−1.65 (0.88)	−7.05	<10^–5^**	151 (45)	−0.95 (0.65)	−4.83	<0.001**

Results of one-sample *t*-tests comparing MMN amplitudes against the signal level at baseline 0 μV at the Fz electrode. Showing mean peak latencies and mean amplitudes with standard deviations (SD). Degrees of freedom (df) for the NH controls = 13; df for CI users = 10. Cases where MMN amplitude diverged significantly from the baseline after Bonferroni-correction (*p* < 0.003) are marked with **.

### Effects of Level and Group on MMN Amplitude

#### Main Effects

**FIGURE 2:**
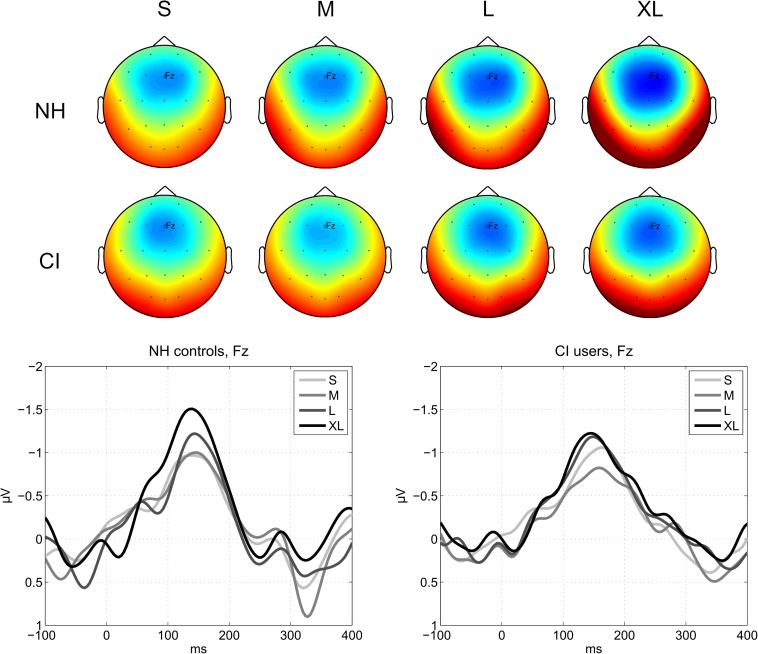
MMN responses to deviant levels. **(Top)** Average MMN scalp topographies measured in a 30 ms time window centered on the global peak in the grand-average waveform at a latency of 147 ms. The colors are scaled from –2 μV (blue) to +2 μV (red). **(Bottom)** Average MMN waveforms for each deviant level and group measured at the Fz electrode.

There was no main effect of Group on MMN amplitude (mean CI users = −1.02 μV, SD = 0.30; mean NH controls = −1.19 μV, SD = 0.34), suggesting that the overall MMN across levels and features did not differ significantly between CI users and NH listeners (Table 2). There was a statistically significant main effect of Level on MMN amplitudes (*S* = −0.97 μV, SD = 0.31; *M* = −0.89 μV, SD = 0.31; *L* = −1.22 μV, SD = 0.50; XL = −1.36 μV, SD = 0.40) [*F*(3,69) = 21.33, *p* < 10^–9^, ${\text{η}}_{\text{p}}^{2}$ = 0.48) (Figure 2 and Table 3). *Post hoc* comparisons confirmed that the effect was driven by a significantly higher MMN amplitude to the XL and L compared to the M and S levels (Table 4).

**TABLE 3:** Analysis of variance.

**Effect on MMN amplitude**	**df**	**df error**	***F***	***p***	**${\text{η}}_{\text{p}}^{2}$**
Level	3	69	21.33	<10^–9^**	0.48
Group	1	23	1.43	0.244	0.06
Level × Group	3	69	2.27	0.088	0.09
Level × Group × Feature	9	207	1.94	0.048*	0.08

**Effect on MMN latency**	**df**	**df error**	***F***	***p***	**${\text{η}}_{\text{p}}^{2}$**

Level	3	69	2.71	0.052	0.11
Group	1	23	1.25	0.276	0.05
Level × Group	3	69	0.84	0.476	0.04
Level × Group × Feature	9	207	0.98	0.461	0.04

**Effect on behavioral hit rates**					

	**df**	***n***	**χ*^2^***	***p***	

Level	3	25	49.54	<10^–9^**	

	**df**	***n***	***U***	***p***	

Group	1	25	34.50	0.015**	

First is shown the effects of Level, Group, Group by Feature, and Group by Feature by Level on MMN amplitude and latency (tested with mixed-effects ANOVA). Finally, the effects of Level (tested with Friedman’s ANOVA) and Group (investigated with the Mann–Whitney *U*-test) on behavioral hit rates are reported. ** indicates significant differences; * marks trends at *p* < 0.05 without correction for multiple comparisons.

**TABLE 4:** *Post hoc* comparisons.

**MMN amplitude**					**Behavioral hit rates**				
**Feature**	**Level**	**XL**	**L**	**M**	**Feature**	**Level**	**XL**	**L**	**M**
**Planned comparisons for each Group, Feature, and Level by Level (corrected α = 0.05/24 = 0.0021)**									
**Normal hearing (*n* = 14)**									
Intensity	L	0.186			Intensity	L	0.014*		
	M	0.008*	0.231			M	0.004*	0.038*	
	S	0.011*	0.125	0.540		S	< 0.001**	0.001**	0.008*
Pitch	L	< 0.001**			Pitch	L	0.317		
	M	< 0.001**	0.764			M	0.039*	0.024*	
	S	< 0.001**	0.422	0.198		S	0.024*	0.024*	0.886
Timbre	L	0.961			Timbre	L	0.317		
	M	0.014*	0.005*			M	0.040*	0.017*	
	S	0.019*	0.043*	0.243		S	0.005*	0.003*	0.065
Rhythm	L	0.636			Rhythm	L	0.285		
	M	0.132	0.147			M	0.141	0.066	
	S	0.002**	< 0.001**	0.003*		S	0.001**	0.001**	0.002**
**CI users (*n* = 11)**									
Intensity	L	0.068			Intensity	L	0.865		
	M	0.002**	0.245			M	0.139	0.153	
	S	0.016*	0.406	0.849		S	0.028*	0.038*	0.344
Pitch	L	0.942			Pitch	L	0.068		
	M	0.915	0.971			M	0.206	0.175	
	S	0.697	0.754	0.677		S	0.943	0.015*	0.235
Timbre	L	0.186			Timbre	L	0.599		
	M	< 0.001**	0.005*			M	0.122	0.191	
	S	0.463	0.402	< 0.001**		S	0.011*	0.028*	0.812
Rhythm	L	0.037*			Rhythm	L	0.414		
	M	0.618	< 0.001**			M	0.038*	0.157	
	S	0.225	< 0.001**	0.235		S	0.007*	0.010*	0.011*

**MMN amplitude**					**Behavioral hit rate**				
**Level**	**XL**	**L**	**M**	**Level**	**XL**	**L**	**M**

***Post hoc* comparisons for main effect of Level (corrected α = 0.05/6 = 0.0083)**							
**All participants (*n* = 25)**							
L	0.043*			L	0.576		
M	<10^–6^**	0.001**			M	0.003**	0.002**		
S	<10^–5^**	<0.001**		0.175	S	<10^–4^**	<10^–4^**		<10^–4^**

**MMN amplitude**					**Behavioral hit rate**				
**Feature**	**Intensity**	**Pitch**	**Timbre**	**Rhythm**	**Feature**	**Intensity**	**Pitch**	**Timbre**	**Rhythm**

***Post hoc* comparisons for Group by Feature (corrected α = 0.05/4 = 0.0125)**									
**All participants (*n* = 25)**									
CI–NH					CI–NH	0.015*	<0.001**	0.058	0.434

*p*-values for (1) the planned comparisons for each Group, Feature, and Level tested by comparing the deviant levels, (2) the main effect of Level, and (3) the potential interaction between Group and Feature. Mean MMN amplitudes for Level differences are compared with paired-samples *t*-tests. For behavioral hit rates, Level and Feature differences are compared using Wilcoxon signed rank tests. ** indicates significant differences; * marks trends at *p* < 0.05 without correction for multiple comparisons.

#### Interactions

Neither the two-way interaction between Group and Feature nor the 3-way interaction between Feature, Level, and Group passed the Bonferroni-corrected threshold α = 0.05/4 = 0.0125 (Table 3).

#### Planned Comparisons

**FIGURE 3:**
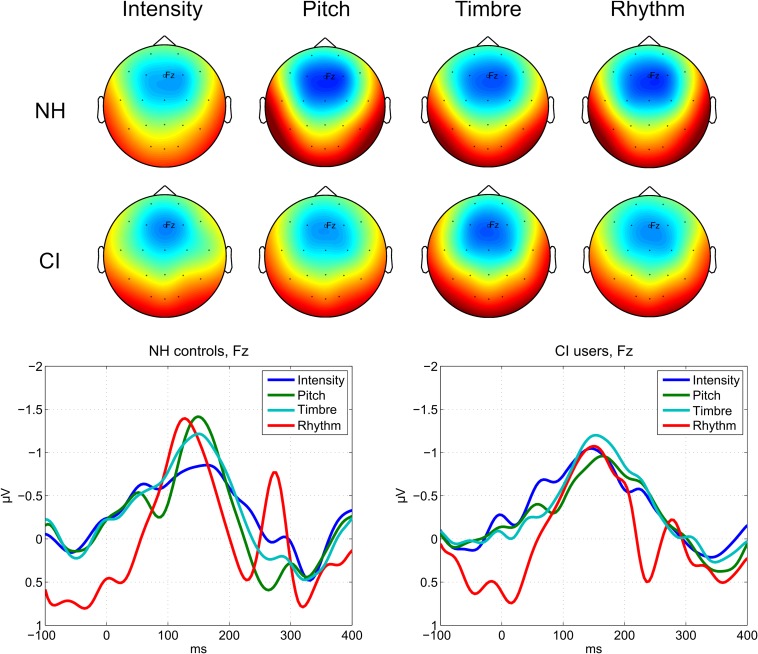
MMN responses to deviants in each auditory feature. **(Top)** Average MMN scalp topographies measured in a 30 ms time window centered on the peaks for each feature. The colors are scaled from –2 μV (blue) to +2 μV (red). NH, normal hearing controls; CI, adult cochlear implant users. **(Bottom)** Average MMN waveforms for each feature and group measured at the Fz electrode.

**FIGURE 4:**
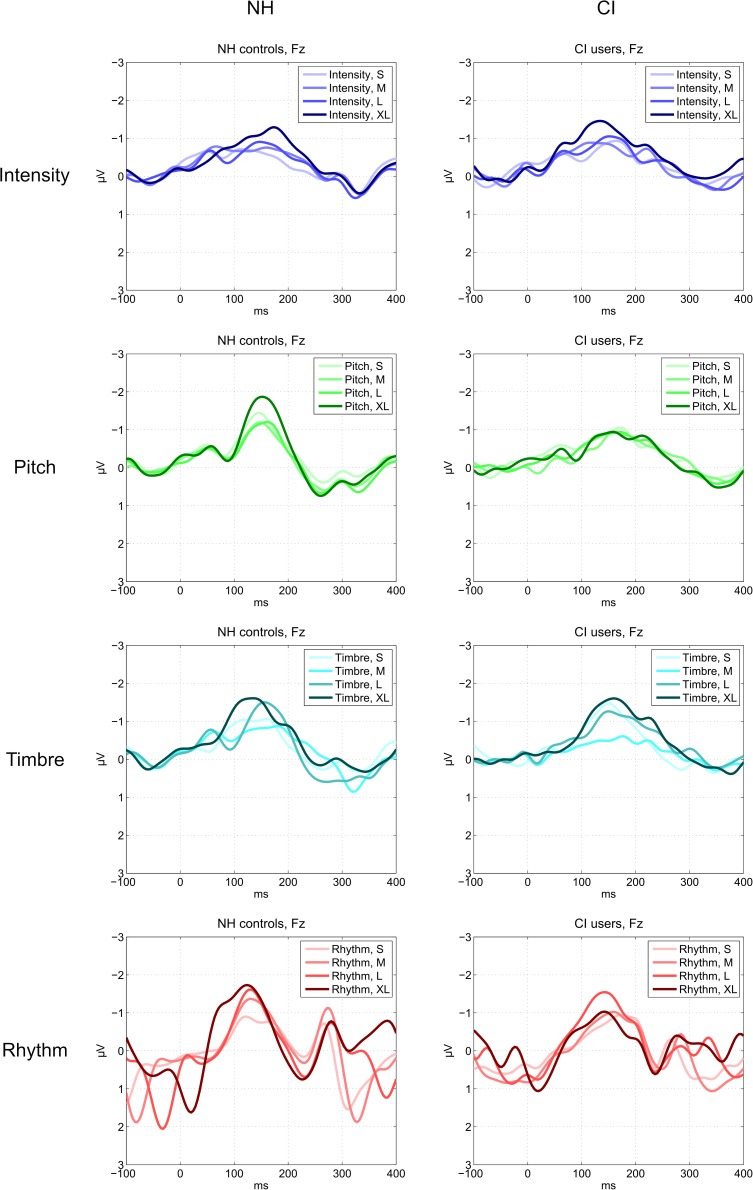
MMN responses to deviants for each feature, level, and group measured at the Fz electrode.

In accordance with our planned comparisons, we here report results of paired samples *t*-tests comparing all levels for each feature within each group. Only results meeting the Bonferroni-corrected significance level at α = 0.05/(4 ^∗^ (3 + 2 + 1)) = 0.05/(4 ^∗^ 6) = 0.0021 are reported here. For full reporting, all results, including trending results at *p* < 0.05 without correction for multiple comparisons, are shown in Table 4. Plots showing difference waves for all deviants and levels are provided in Figures 3, 4.

#### Intensity

Normally hearing listeners showed no differentiation between any levels of the Intensity deviant. In CI users, there was a significantly larger MMN amplitude to the XL compared to the M intensity deviants.

#### Pitch

In NH listeners the MMN amplitude to the XL deviant was significantly larger than that of the L, M, and S deviants. The CI users did not show any significant differences in their MMN amplitudes between pitch levels.

#### Timbre

In the NH group, no significant differences were found between any levels of the Timbre deviant. CI users demonstrated a significantly higher MMN amplitude to the XL compared to the M deviant. By contrast, CI users’ MMN amplitude to the S deviant was significantly higher than that elicited by the M deviant.

#### Rhythm

Normally hearing listeners’ MMN amplitudes to rhythm deviants were significantly larger for the XL and L deviants compared to S deviants. In CI users, the MMN amplitude was significantly higher to the L deviant than to M and S deviants.

### Effects of Level, Feature, and Group on MMN Latency

#### Main Effects

There was no main effect of Group on MMN latency (mean CI users = 168 ms, SD = 11; mean NH controls = 162 ms, SD = 14), suggesting that the overall MMN latency across levels and deviants did not differ significantly between CI users and NH listeners (Table 3). Also, there was no main effect of Level on MMN latency (Table 3).

#### Interactions

Neither the two-way interaction between Group and Feature nor the three-way interaction between Feature, Level, and Group were significant for MMN latency.

### Behavioral Discrimination Scores

#### Performance vs. Chance

One-sample Wilcoxon signed rank tests showed that for NH controls hit rates were significantly higher than chance level (33%) for all features and levels, except for the Intensity S and M, Timbre S, and Rhythm S deviants (Table 5). CI users exhibited a high degree of individual variability, scoring significantly above chance level only for the Rhythm XL, L, and M deviants as well as the Timbre L and XL deviants (Bonferroni-corrected threshold: α = 0.05/16 = 0.003) (Table 5).

**TABLE 5:** Behavioral hit rates.

**Feature**	**Level**	**NH controls**			**CI users**		
		**Median hit rate in % (range)**	***Z***	***p***	**Median hit rate in % (range)**	***Z***	***p***
Intensity	S	33.0(17−50)	1.05	0.293	33.0(0−67)	−0.54	0.592
	M	58.5(17−83)	2.69	0.007*	33.0(0−83)	−0.27	0.789
	L	75.0(33−100)	3.12	0.002**	50.0(17−83)	2.05	0.040*
	XL	100.0(50−100)	3.37	<10^–3^**	50.0(0−100)	1.78	0.075
Pitch	S	100.0(50−100)	3.37	<10^–3^**	66.7(17−100)	2.41	0.016*
	M	100.0(50−100)	3.37	<10^–3^**	83.0(17−100)	2.68	0.007*
	L	100.0(67−100)	3.64	<10^–3^**	100.0(33−100)	2.93	0.003*
	XL	100.0(67−100)	3.56	<10^–3^**	67.0(0−100)	2.43	0.015*
Timbre	S	83.0(17−100)	2.94	0.003*	66.7(33−100)	2.86	0.004*
	M	91.7(33−100)	3.28	0.001**	66.7(17−100)	2.69	0.007*
	L	100.0(100−100)	3.74	<10^–3^**	83.0(50−100)	2.97	0.003**
	XL	100.0(83−100)	3.64	<10^–3^**	100.0(50−100)	2.99	0.003**
Rhythm	S	50.0(17−100)	2.38	0.017*	50.0(17−100)	2.42	0.016*
	M	100.0(50−100)	3.40	<10^–3^**	83.0(50−100)	2.98	0.003**
	L	100.0(83−100)	3.56	<10^–3^**	100.0(33−100)	3.03	0.002**
	XL	100.0(50−100)	3.56	<10^–3^**	100.0(83−100)	3.21	0.001**

Behavioral hit rates exceeding the chance level for correct answers at 33.3%. Median hit rates are depicted with ranges (min–max). Degrees of freedom (df) for the NH controls = 13; df for CI users = 10. Hit rates significantly greater than chance level after Bonferroni-correction (*p* < 0.003) are marked with **; those significant without correction (*p* < 0.05) are marked with *.

#### Main Effects

The Mann–Whitney *U*-test comparing the group scores across Features and Levels revealed an overall significant difference between groups, with lower hit rates for the CI-users (median: 75%) than the NH controls (median: 100%) [*U*(25) = 34.50, *p* = 0.015, *r* = 0.49]. Furthermore, the Friedman’s ANOVA showed a main effect of Level [χ^2^(25) = 49.53, *p* < 10^–9^, *r* = 0.69] (Table 3). *Post hoc* comparisons confirmed that the effect was driven by significantly higher hit rates for the XL and L compared to the M and S levels and for the M compared to the S level (Table 4).

#### Interactions

**FIGURE 5:**
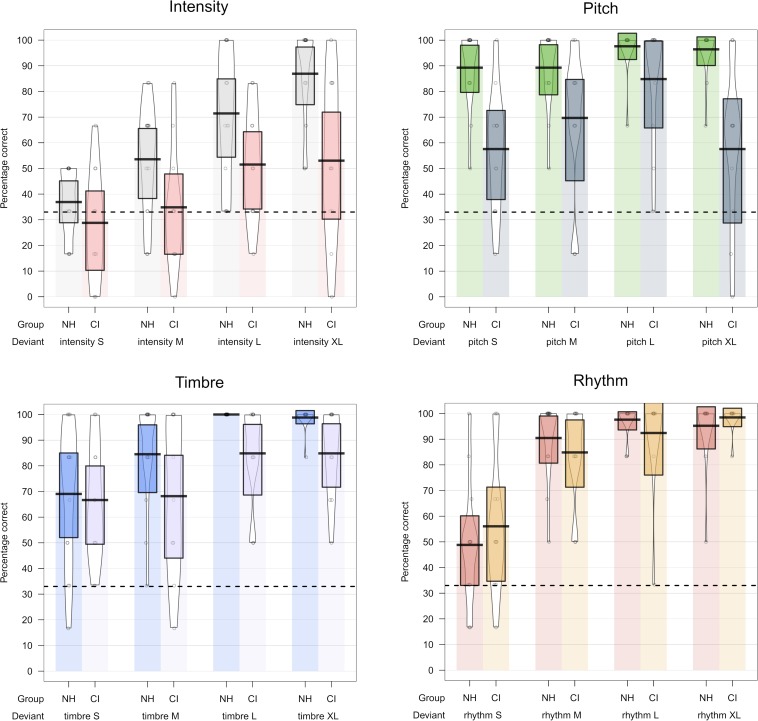
Violin plots showing behavioral hit rates for each feature, level, and group. Dotted line indicates chance level.

As already mentioned, the standard Friedman’s ANOVA does not include tests of interaction effects, and we therefore report *post hoc* comparisons for the potential Group by Feature interaction in Table 4. They indicated that the group difference was mainly driven by significantly lower hit rates in the CI group compared to the NH group for the pitch deviants (Table 4 and Figure 5).

#### Planned Comparisons

In accordance with our planned comparisons, we here report results of one-sample Wilcoxon signed rank tests comparing all levels for each feature within each group. We only report results passing the Bonferroni-corrected significance level at *p* = 05/(6 ^∗^ 4) = 0.0021 here. All results, including trending results at *p* < 0.05 without correction for multiple comparisons, are shown in Table 4 for full reporting. Violin plots illustrating the behavioral results are provided in Figure 5.

#### Intensity

In the NH group, the intensity XL and L deviants resulted in significantly larger hit rates compared to the S deviant. No significant differences between any levels were observed in CI users.

#### Pitch

The NH group showed a ceiling effect with no significant difference between any levels. In CI users, no significant differences in hit rates were observed.

#### Timbre

For timbre, none of the two groups showed any significant differentiation of any of the deviant levels.

#### Rhythm

Normally hearing participants showed significantly higher hit rates for the XL, L, and M compared to the S rhythm deviant. CI users did not show any significant differentiation in terms of hit rates.

### Correlation Between MMN Amplitude, Behavioral Scores, and Clinical and Music Appreciation Factors

We found no statistically significant effects of any clinical or music appreciation factors on neither the CI users’ MMN responses nor their behavioral hit rates. A weak positive relationship was found between age at hearing loss and strength of MMN amplitudes for the rhythm (*p* = 0.026, uncorrected) and timbre (*p* = 0.071, uncorrected) deviants, indicating that a larger MMN was associated with later age at hearing loss.

## Discussion

This experiment assessed the cortical and behavioral discrimination of musical features in adult CI users and NH controls. The electrophysiological measurements were performed using a multifeature MMN-paradigm presenting four musical features at four levels of deviation magnitude in a “no-standard”-design. In accordance with our hypothesis, the paradigm elicited robust MMN responses to all deviation levels in both NH controls and CI users. Furthermore, across participants, the results showed an overall relationship between MMN strength and deviation magnitude; the larger the deviation, the stronger the MMN response. Finally, contrary to our hypothesis, neither the overall MMN amplitudes nor latencies of the CI users were significantly different from those of the NH group. The findings extend previous multi-feature MMN studies indicating that CI-recipients using present-day speech processing technology may be capable of detailed discrimination of musical sounds even when presented in a complex context (Sandmann et al., 2010; Torppa et al., 2012; Petersen et al., 2014; Timm et al., 2014; Hahne et al., 2016).

The CI-MuMuFe MMN-paradigm constitutes an unprecedented level of complexity in MMN research of CI-users. Nevertheless, our results indicate that the paradigm is both accurate and feasible and may provide strong evidence of CI users’ musical discrimination abilities and thresholds as a tool for objective measurements of music discrimination. The use of the paradigm could be of clinical relevance, because it allows for detailed measurement of auditory discrimination abilities in CI patients within a time frame sufficiently short to avoid fatigue and demotivation (Näätänen et al., 2004). Furthermore, in a clinical context, the CI MuMuFe-paradigm could be used as an objective tool for assessment of auditory rehabilitation after CI, e.g., auditory verbal therapy (Sandmann et al., 2010; Rahne et al., 2014) or music training. Especially in the case of young children who receive implants before language acquisition and in whom subjective responses are difficult to interpret, MMN responses might provide useful information regarding the development of auditory functions (Sharma, 2006). To fully qualify for clinical use, however, it is important to improve the analytical methodologies such that MMN measures can be estimated not only at the group level but also in individuals (Bishop and Hardiman, 2010). This is further substantiated by the high degree of variance in the individual MMN traces shown in Supplementary Figure 2.

### Behavioral Measurements

On average, the CI users scored significantly below the NH listeners in the behavioral discrimination of the four types of music deviants. This was particularly true for Pitch and Intensity, whereas the CI users’ detection of changes in Rhythm was not significantly different from that of their NH counterparts (Table 4). This confirms previous reports, showing that CI users score significantly below NH controls in pitch-related tasks such as melodic contour recognition but usually perform at nearly comparable levels on rhythmic tasks (Gfeller et al., 2007; Drennan and Rubinstein, 2008; Jiam, 2017). As for MMN, the behavioral results showed a significant effect of level across groups. The effect was most prominent in the NH listeners’ detection of changes in Intensity and Rhythm while the CI group showed no significant effect of level for any features. However, as also suggested in the violin plots in Figure 5, the CI-users in general showed trends toward scoring more accurately when presented with larger changes of the different features.

### Individual Variation

As already noted, the CI users’ behavioral performance was characterized by a large amount of variation. While some CI users scored at or below chance, others were able to achieve 100% correct scores for all deviant levels except the two lowest levels of the Intensity deviant. This gross variability in performance is a well-known phenomenon in CI-research and may reflect differences in the patients’ history of hearing loss (Blamey et al., 2013) and, in this case, musical background. However, our correlation analyses did not identify any significant clinical or music-related factors predictive of either neurophysiological or behavioral performance. The only exception was age at hearing loss which tended to be positively associated with MMN amplitude, indicating that loss of hearing at a young age may hamper the development of fine-tuned auditory processing.

Another potential source of variation is age. However, despite a wide span of age (35–80 years) among the CI-users, age showed no significant relation with any outcome measures. The difference in mean age between the two groups with NH controls being slightly (albeit non-significantly) older than the CI users might represent a possible limitation. As aging can affect the MMN negatively (Schiff et al., 2008), it is fair to speculate that this might to some degree contribute to the lack of difference in MMN amplitude between the two groups. We will in a subsequent article report on the potential effect of aging on the MMN as measured with the CI-MuMuFe paradigm from a separate study involving also a group of NH young adults.

### MMN and Behavior Relationship

So why were CI users’ behavioral performances poorer than NH listeners’ when their MMNs were not significantly different? The presence of significant MMNs indicates that at the early pre-attentive stages of sound processing, the brain is able to detect the subtle sound differences. However, in the active attended listening task, other factors than perceptual sensitivity might influence the performance (Bishop, 2007; Bishop and Hardiman, 2010). For CI users, making meaning of complex sounds constitutes a great demand for cognitive resources and listening effort (Giraud et al., 2000; Hughes et al., 2018). Thus, in a task involving unfamiliar sounds and with the absence of visual cues, some degree of fatigue or unsustained attention may explain this inconsistency between neurophysiology and behavior.

It is also important to point out that MMN and behavioral testing performance often fail to correspond in a strict one-to-one fashion; strong MMNs are not necessarily associated with higher scores (Bishop and Hardiman, 2010; Ortmann et al., 2017). This is also indicated in this study by the lack of significant correlations between MMN amplitudes and hit rates, which thus lends further support to the notion that a group difference at the behavioral level may not necessarily correspond to a group difference at the neurophysiological level.

### Discrimination vs. Music Appreciation

On average, the CI users were able to detect some of the subtle changes incorporated in the paradigm, albeit more so at the neural than at the behavioral level. It should be emphasized, however, that this does not necessarily warrant music appreciation. As also suggested by the lack of a relationship between self-reported music appreciation and discrimination skills, other factors may play a significant role in the degree to which CI users tend to like music. Whereas many CI users experience reduced music enjoyment after implantation (Mirza et al., 2003), some studies show that for some CI users enjoyment of music is not hindered despite lack of ability to perceive pitch and timbre (Looi et al., 2012), and that especially rhythm and lyrics are important components of enjoyment (for a review see Riley et al., 2018). It is beyond the scope of this study to deal with this interesting research avenue in further detail. However, future research could potentially employ the CI-MuMuFe paradigm to further elucidate the role of different musical features in CI users’ music appreciation.

### Features

#### Intensity

Intensity (or loudness) contributes to the experience of dynamics and is an important prerequisite for the full extent of music enjoyment. In the present experiment, the changes in intensity were quite subtle, as reflected both in the very low hit rates and the relatively weak MMN responses observed in the NH listeners. Nevertheless, CI users exhibited cortical responses that reflected the level hierarchy. This was quite surprising, since, because of necessary compression of the sound signal, the dynamic range of the CI is often limited to 6–30 dB, as compared to the potential NH range of 120 dB (Shannon, 1983; Moore and Moore, 2003; Zeng, 2004).

A previous MMN-study by Sandmann et al. (2010) tested discrimination of intensity presented in decremental steps of 4 dB and found a significant MMN only for the largest level in NH and for the second largest level in CI. Since the only difference between the two studies is the design of the paradigm – odd-ball vs. arpeggiated triads – explanations for this discrepancy could be either improved sound processing technology or differences in methodological approaches related to recording or analysis of the ERPs.

It would be fair to argue that presenting intensity in decremental steps only paints an incomplete picture of perception of dynamic changes. The rationale for this one-way approach, however, is to avoid exceeding the sound level of 65 dB and the potential risk of distortion at the higher levels. In their study with CI children, Torppa et al. (2012) presented both decremental and incremental intensity deviants but found MMN responses only for the decremental deviants. The authors speculated that the lack of response could be linked either to CI-listeners’ limited processing of stronger loudness levels or to the sound processor’s automatic gain control. This possible limitation will be further explored in our ongoing investigation of CI users’ neurophysiological responses to a new “Free-listening”-paradigm in which the participants are exposed to real music (Poikonen et al., 2016).

#### Pitch

Pitch perception is crucial for identifying melodic contour both in relation to music and to language. Several studies have concluded that CI users’ perception of pitch is poor and that some CI users may need intervals of several semitones to identify a change of pitch (Looi et al., 2012; Limb and Roy, 2014). Interestingly, the CI users in the present study showed a robust MMN response not only to the larger but also to the smallest pitch change of one semitone. This is consistent with Hahne et al. (2016) who found that whereas a pitch deviant of +1 semitone elicited a “good MMN potential”, a −24 ct (a quarter of a semitone mistuning) did not. The authors concluded that “the real performance optimum of pitch discrimination of the CI stimulation might be still somewhat below 1 semitone.” In that perspective, taking also the CI users’ undifferentiated neural discrimination of levels of pitch change into account, it may be worth considering including a quarter-tone (half semitone) pitch deviant in a future revision of the CI-MuMuFe. Such an adjustment might also to some extent reduce the ceiling effect found in the NH controls’ behavioral performance.

The CI users’ behavioral discrimination of the pitch deviant showed a large variability, failing to reach a within-group consistent above-chance level. Nevertheless, whereas the MMN responses did not reflect effects-of-magnitude, tendencies in the behavioral results suggested that CI-users might obtain higher discrimination accuracy for the L compared to the S and M deviation levels (Figure 5 and Table 5). This may reflect the difference between the early pre-attended change detection represented by the MMN and the attended, conscious detection and perception as measured in the behavioral task. The large variance observed in the behavioral identification of the XL 8-semitone-change, exhibiting floor as well as ceiling effects, is difficult to interpret. We speculate that individual differences in both the CI-processing strategies and auditory profiles may be the cause of this inconsistency.

#### Timbre

Consistent with previous reports, timbre deviants elicited robust MMN responses in both groups (Petersen et al., 2014; Timm et al., 2014; Hahne et al., 2016). Furthermore, the neural discrimination of the four deviant levels in general reflected the deviation magnitude. In CI users, however, the small “bright” piano variation elicited an MMN response that was significantly larger than the medium “blues” variation. This unexpected difference in automatic detection could be due to extraction of envelopes triggered by the richer representation of higher frequencies in the activation of electrodes, as also illustrated in Supplementary Figure 5.

While the selection of the trumpet and electric guitar deviants was based on experience gained from previous experiments, the two smaller deviants were created from the logic of making slight variations of the standard piano sound. As can be seen in Supplementary Figure 5, that logic was not totally wrong. The bar plot top right shows the increasing amount of spectral energy that differentiates the standard sound from the deviant sounds. However, when running the sounds through a CI simulator, as shown bottom right, the “bright” piano sound clearly exhibits a stronger spectral envelope than the “blues” variation. The phenomenon is a fine exemplification of how different electric hearing is from normal hearing. A future revision of the paradigm should consider taking this observation into account by reversing the two in the level hierarchy.

Of interest in this context is a study on timbre perception in adult CI users using behavioral performance as a model for individually adapted MMN stimuli (Rahne et al., 2014). Instead of instrument sounds, the paradigm presented synthesized tones with varying relationship between the fundamental and a spectrum of harmonics. The authors concluded that MMN responses reflected the individual threshold for automatic detection of timbre changes.

Even though the CI users here exhibited differentiated neural detection of changes in timbre, it is important to note that this may not necessarily reflect ability to distinguish or recognize instruments. This task is notoriously challenging for CI users (Heng, 2011; Looi et al., 2012; Limb and Roy, 2014), although effects of training have been reported (Driscoll, 2012; Petersen et al., 2012; Jiam et al., 2019). What we show is the neurophysiological and behavioral capability to identify subtle changes in the “color” of a sound which is a prerequisite for possible further training of this skill.

#### Rhythm

Whereas the spectral resolution of the CI signal is low, the temporal resolution is high as reflected in near normal rhythm discrimination reported in behavioral studies (Limb et al., 2010), MMN studies (Petersen et al., 2014; Hahne et al., 2016), as well as in effect of targeted rhythm training (Petersen et al., 2012). Our results confirm this, showing CI performance that is not significantly different from that of the NH group and significantly better for rhythm than for the other three deviants (Table 4 and Figure 5).

The four rhythm deviants in the paradigm follow the logic of beat subdivision, such that the 26 ms anticipation in a musicological concept is a 64th note syncopation whereas the largest deviant equals a dotted 16th-note syncopation (Figure 1). Both NH and CI listeners’ discrimination of the rhythm deviants tended to follow this musical logic: the shorter the displacement of the third note the more difficult the detection.

As a single exception, the CI users showed the strongest MMN response to the second largest rhythm deviant and not the largest (Table 2). This could be explained by the very short distance (50 ms) between the second and third note which may be perceived as a merging of the two notes. So, even though the two notes are three semitones apart, they may be perceived as the same note because of the CI’s poor representation of pitch. By contrast, in their attended behavioral detection of the rhythm deviants, the CI group detected the largest deviant most accurately (Figure 5). However, in that task the requirement is to detect which of three patterns is different. Thus, the largest rhythm deviant is clearly identifiable because of the omission of the third tone at the expected position.

### Methodological Considerations

#### The Standard Response

In the original paradigm from Vuust et al. (2011), a standard pattern was played in between every deviant pattern. Thus, the ERP elicited from the third tone in the standard configuration could be subtracted directly from the response to the third tone in the deviant pattern, eliciting the MMN response. In the no-standards paradigm from Kliuchko et al. (2016) the standard pattern was omitted which meant that the third tone was never a standard. Consequently, no direct comparison between the standard and deviant response was possible. Instead, the standard response was defined as the response to the first, second, and fourth tone of the Alberti bass pattern, because these tones never occurred as deviants. An average between the first, second, and fourth tone is a compromise between several other less ideal standard responses in an attempt to mimic the relatively stable and neutral response to the third tone obtained in the original paradigm. The less ideal standard responses (the first, the second, or the fourth tone, respectively) are all confounded by the MMN response or by N1 enhancement in their baseline or in their post-stimulus time window, which is visualized in Supplementary Figure 3. For clarity it is important to point out that since the same standard response is subtracted from each of the compared 16 deviants, the statistical differences observed for the within-subject factors of Level and Feature could not have been affected by the choice of standard response.

See the Supplementary Material for a more in-depth discussion of the different scenarios for selecting an appropriate standard response.

#### The Rhythm Deviant and Its Baseline Correction

The rhythm deviant is different from the other deviants as it actually consists of two deviants in one. First, it is a duration deviant because the second note in a rhythm deviant sequence is shortened 26, 52, 103, and 155 ms, respectively. Second, it is a rhythm deviant since the third note is thereby presented earlier than expected. The fourth note, however, is unchanged and occurs at the usual time because the third note of the rhythm deviant sequence is prolonged accordingly.

The epochs are centered around the prolonged third note to best capture the mismatch response to the rhythm deviant and thereby compare the individual rhythm deviants. However, as the second note becomes shorter, the P50 response occurs closer to the onset of the third note, and in the case of the XL deviant, the P50 response to the shortened note actually begins when the third note has its onset. This presents a challenge with regard to baseline correction because the conventional 100 ms baseline window preceding the third note is hereby contaminated with P50 responses to the preceding note to varying degrees depending on the extent of the shortening, and thus especially so for the L and XL deviants as illustrated in Supplementary Figure 4.

The contamination of the conventional baseline window thus co-varies with the four levels of the rhythm deviants, which constitute a factor in the statistical analyses. Therefore, we opted for the 100 ms period preceding the shortened second note as baseline correction window for the rhythm deviants. The ongoing activity in this time window was unaffected by the varying overlap between P50 responses and baseline windows caused by the shortening of the second note, and thus served as a good estimate of the ongoing background activity prior to the rhythm deviants (see the Supplementary Material for more details on the choice of baseline window for the rhythm deviants).

#### Speaker vs. Cable

In the present study, CI users were presented with the sound stimuli through a direct audio input cable rather than listening via loudspeakers. This allows for control of which sound inputs are presented to the participants and eliminate confounding factors such as residual hearing. Some challenges, however, are associated with this method. First, present-day CIs are quite small, leaving no room for an audio input port. Thus, for most of the CI users, a spare processor had to be programmed with their personal mappings. This obviously may be the source of some experimental uncertainty as well as participant concern. The newest generation of CI processors, however, provide a fast, wireless connection which may eventually eliminate this problem.

Some of the CI users had bilateral CIs and were forced to choose their best performing ear for the tests. Both for them and for the bimodal listeners, the monaural stimulation represented a listening situation that was less satisfactory and quite far from what they were used to. We can only speculate the degree to which this affected their performance. We would argue that even though there is a trade-off when presenting the stimuli directly it represents the most optimal basis for a fair comparison and standardizes one factor in a population already characterized by a multitude of profiles.

#### Sound Intensity

Due to quite varying degrees of tolerance of the volume of the stimuli, we were unable to maintain a perfect match in the sound intensity between CI-participants. This may introduce a possible variance in the recorded EEG data. However, because the participants had to listen to the stimuli for 32 min and were asked not to focus on the sound, we considered it most important that the sound level was tolerable for the individual participant. The MMN is affected by attention, which means that if an individual was disturbed by the stimuli, this might affect the results. Because the perceived loudness with a CI depends on both the chosen program and the individual settings, a direct comparison of sound levels between CI users is virtually impossible. Thus, we conclude that the individual comfortable level is the most optimal way to set the intensity level. Measuring the intensity of the sound coming into the CI is possible by connecting the implant to a software system for visual inspection. However, one thing is what can be seen objectively on a screen, another thing is what is subjectively perceived by the participant.

## Summary and Conclusion

Our findings confirm and expand previous reporting on adult CI-users’ music perception abilities. Despite degraded representation of spectral fine structure in the CI-signal, CI-users exhibited MMN-responses to changes in basic features of music that were significant and not significantly different from those of NH controls. Both groups showed MMN strength that was in alignment with the deviation magnitude. In CI users, however, discrimination of pitch levels remained undifferentiated. CI users’ behavioral performance was significantly below that of the NH group, mainly due to poor pitch discrimination. Although no significant effects were found, CI users’ behavioral results tended to be in accordance with deviation magnitude, most prominently manifested in discrimination of the rhythm deviant.

The findings indicate that the new MuMuFe paradigm can effectively estimate musical discrimination abilities and thresholds in CI users. Furthermore, the large heterogeneity of the CI-users tested in the present study suggests that the paradigm has a promising potential for assessing a wide range of perceptual profiles. Thus, the paradigm may be a valuable tool in measurements of the effect of training or in studies which examine neural plasticity following CI. Furthermore, the CI MuMuFe may have clinical relevance with a potential of evaluating thresholds and limits in follow-up procedures, e.g., in young children for whom subjective measurements are difficult to interpret. Future studies should investigate the possibility of applying the paradigm with the purpose of assessing discrimination skills not only at the group level but also at the individual level.

## Data Availability Statement

The datasets generated for this study are available on request to the corresponding author. Due to the EU General Data Protection Regulation (GDPR) which came into force in 2018 the clinical dataset cannot be made publicly available; it can, however, be obtained by individual researchers upon individual research data sharing agreements.

## Ethics Statement

This study was carried out in accordance with the recommendations of the Research Ethics Committee of the Central Denmark Region. All subjects gave written informed consent in accordance with the Declaration of Helsinki. The protocol was approved by the Research Ethics Committee of the Central Denmark Region.

## Author Contributions

BP, PV, EB, and SR contributed to the conception and design of the study. BP and AA conceived the paradigm and the behavioral test and created all stimuli, and wrote the first draft of the manuscript. AA organized and carried out the recruitment and tests. NH, AH, and MD processed the EEG and behavioral data and performed the statistical analyses. FM provided the technical assistance with programming of spare processors and information about CI participants. NH and AH wrote the sections of the manuscript. All authors contributed to the manuscript revision, and read and approved the submitted version of the manuscript.

## Conflict of Interest

The authors declare that this study received funding from Oticon Medical. The funder had the following involvement with the study: Idea for and contribution to design of study. All authors declare no conflict of interest. The reviewer PV declared a shared affiliation, with no collaboration, with one of the authors, EB, to the handling Editor.

## Footnotes

1 www.beltonehearingtest.com/dk1/
