# Supplementary material for: The CI MuMuFe – A New MMN Paradigm for Measuring Music Discrimination in Electric Hearing
Source: Front Neurosci. 2020 Jan 23;14:2. doi: 10.3389/fnins.2020.00002 (PMC6990974; doi:10.3389/fnins.2020.00002)
Supplement: Supplementary file 1 [file Data_Sheet_1.pdf]

Supplementary Material

1 Deviant and standard waveforms at the Fz electrode with 95% confidence intervals

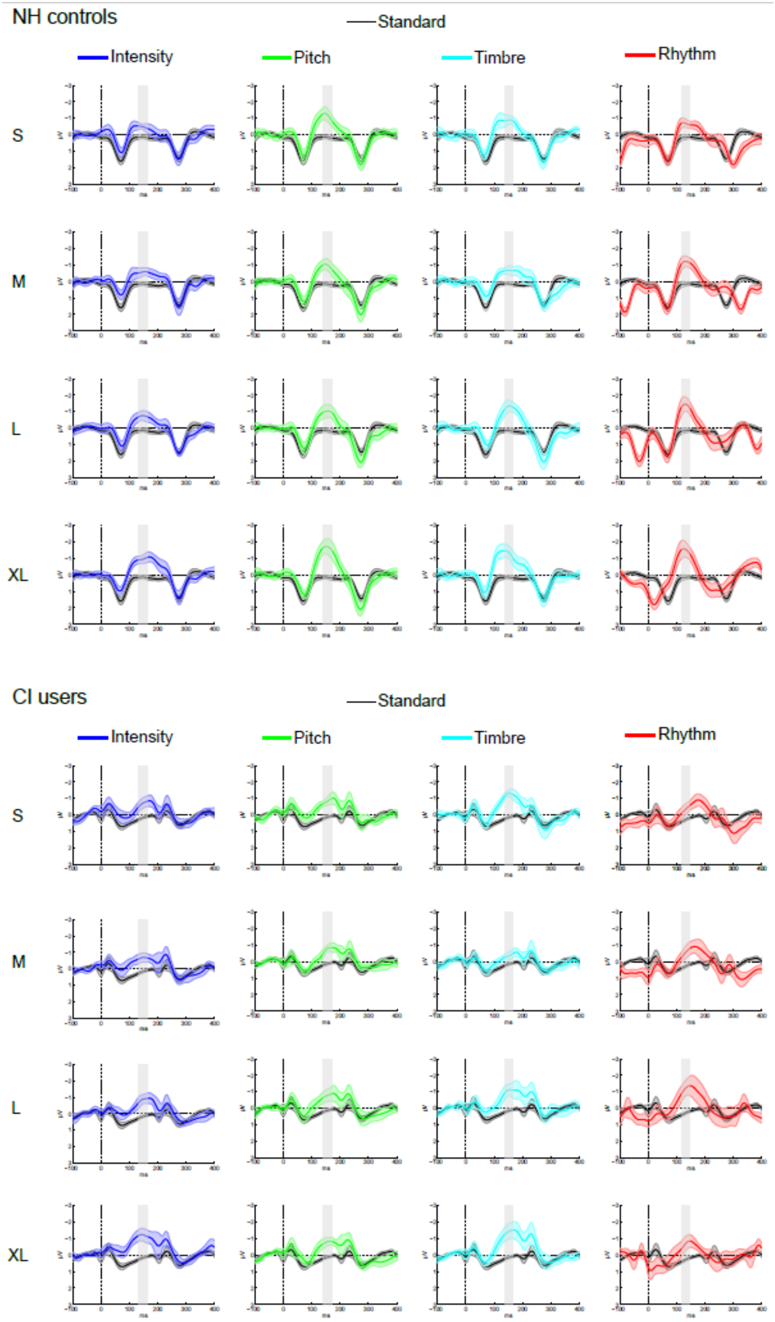

Supplementary Figure 1. Standard and deviant response waveforms. Standard (black) and deviant (colored) waveforms are shown as measured at the Fz electrode. The dashed vertical line marks the onset of the standard or deviant tone at 0 ms. The grey bars indicate the time window applied for the measurement of the MMN amplitudes. The shades around the curves shows the 95% confidence intervals.

2 Single-subject difference waves with group means and 95% confidence intervals.

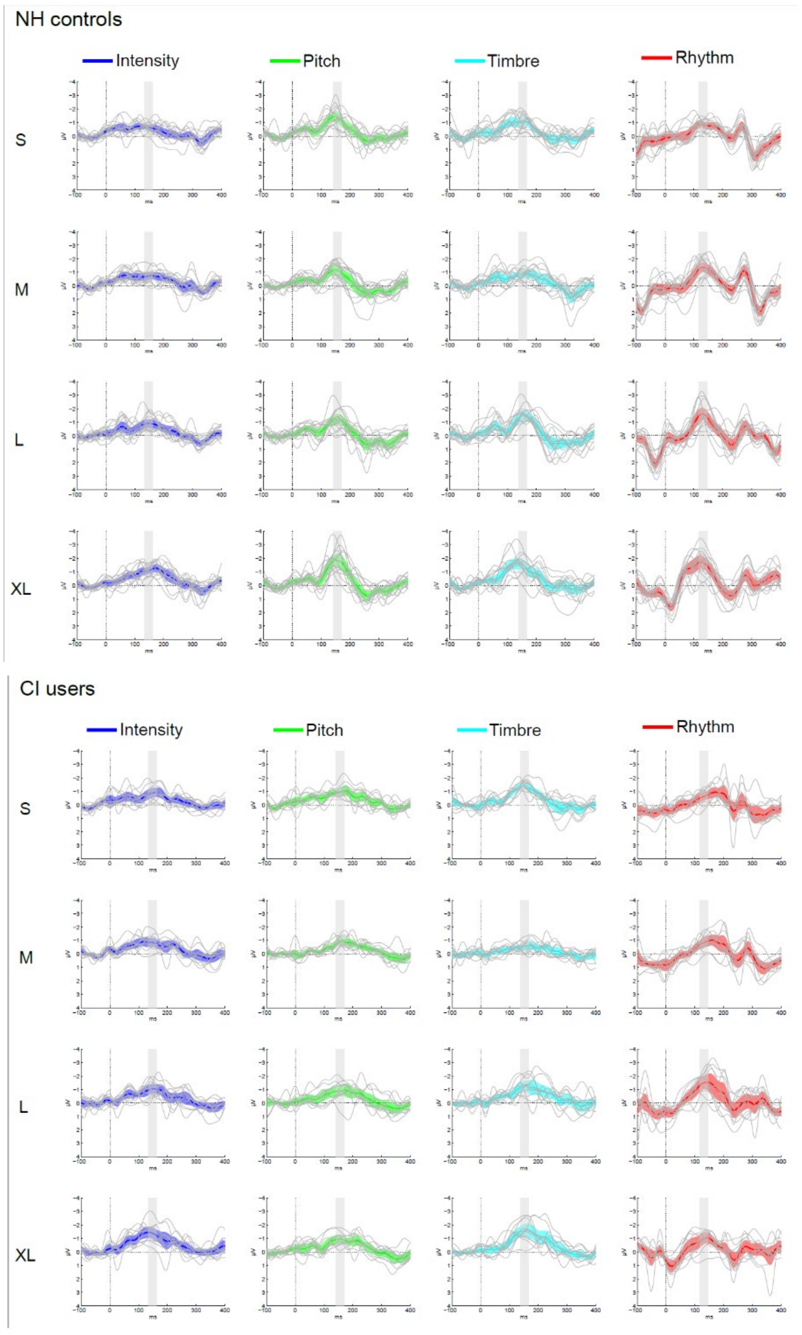

Supplementary Figure 2. Single-subject difference waves with group means. Top NH controls; bottom CI users. Group mean MMN difference waveforms are shown for each feature (colored) along with single subject MMN difference waveforms (thin grey) as measured at the Fz electrode. The dashed vertical line marks the onset of the deviant tone at 0 ms. The grey bars indicate the time window applied for the measurement of the MMN amplitudes. The coloured shades shows the 95% confidence intervals for the group means.

3 Optimal standard response in the no-standard MuMuFe paradigm

The best available standard response waveform was obtained by averaging the evoked responses to the first, second and fourth note in the Alberti bass pattern, as in the previous studies applying the “no-standard MuMuFe” paradigm. The averaged standard waveform was then subtracted from each deviant response (i.e., each third note) to identify the MMN.

However, when selecting which standard response to use as the best available standard response in the no-standard MuMuFe paradigm, different challenges arise. If one selects the ERP waveform to only the first note as the standard, it would contain what seems to be an enhanced N1 response (as evident in Supplementary Figure 3, red curve) which would diminish the early part or most of the MMN component when this enhanced N1 response is subtracted from the negative deflection in the deviant response. If selecting the ERP to only the second note, the enhanced N1 response from the response to the first note overlaps with the 100 ms baseline preceding the second note, causing a biasing of the baseline with the opposite effect of the N1 enhancement when selecting only the first note (see Supplementary Figure 3, bright green curve). Moreover, the 100 ms baseline preceding the fourth note would be similarly biased as it overlaps with the MMN response from the third note (see Supplementary Figure 2, blue curve). By using as the standard waveform, the average across ERPs to the first, second and fourth notes, the N1-enhancement in the ERP to the first note counteracts the effects of the N1-enhancement and the MMN in the baselines of tones 2 and 4, respectively, thus minimizing the overall bias (see Supplementary Figure 3, black curve).

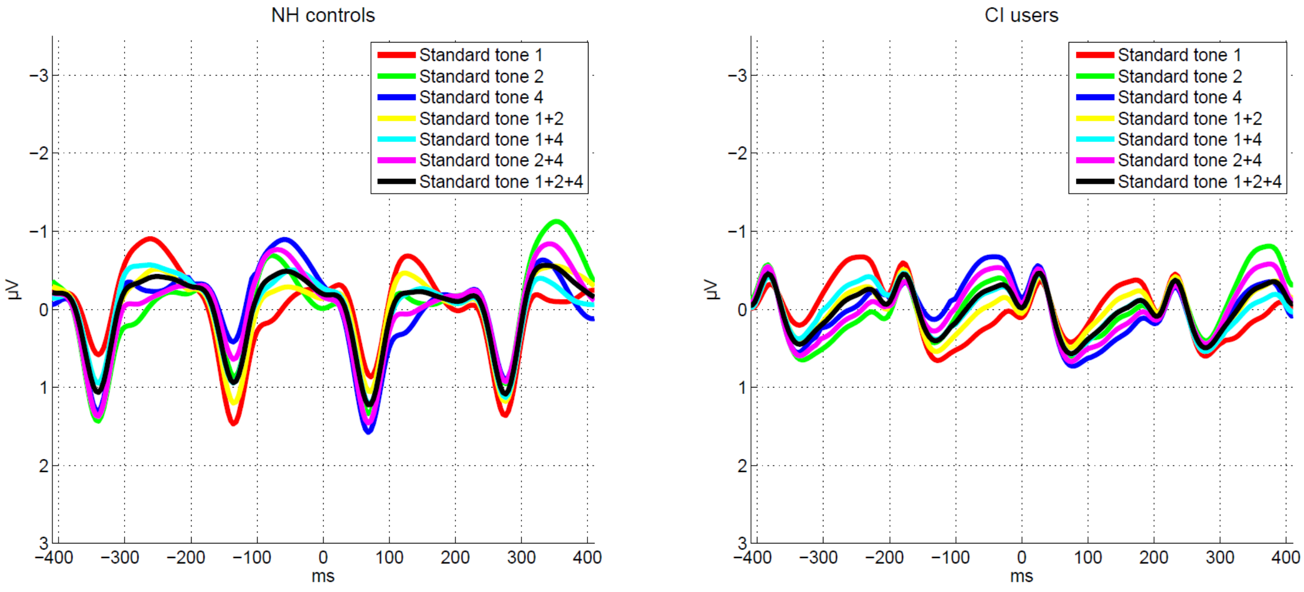

Supplementary Figure 3. Optimal standard waveform. Showing the standard tone responses waveforms for each possible combination of standard tones within the Alberti-bass pattern. When more tones are included the average waveform for these tones is applied. The onset of the tone(s) indicated in the legend above the curves starts at 0 ms.

4 Baseline correction for the rhythm deviant waveforms

A conventional baseline correction approach employing the 100 ms preceding the onset of the rhythm deviant as the third note would include different aspects of the neural processing of the gradually shortened second note depending on the deviant level (see Supplementary Figure 4). In other words, such an approach would confound the comparison of the different levels of the rhythm deviants due to different degrees of the P50 response occurring in this conventional baseline window as a direct effect of the rhythm level manipulation (i.e., the shortening of the preceding note, Supplementary Figure 4, left). To solve this issue of baseline contamination, the rhythm deviant responses were baseline corrected in relation to the 100 ms preceding the shortened second note whereby the baseline period was not directly affected by differences in the P50 response caused by the level manipulation (see Supplementary Figure 4, right).

In relation to the onset of the rhythm deviant: a baseline correction of -305: -205 + 26 ms was applied for the S rhythm deviant, -305: -205 + 52 ms for the M rhythm deviant, -305: -205 + 103 ms for the L rhythm deviant, and -305: -205 + 155 ms for the XL rhythm deviant.

Because the paradigm is so fast and the pauses between the notes are very short (5 ms), the baselines for the other three types of deviants are, of course, also affected by brain responses from the preceding note, e.g. an MMN response or an N1 enhancement as we discuss with respect to the selection of a suitable standard response. However, such effects are rather constant for all levels of the different deviants, and thus do not confound the level comparison directly. It is thus the nature of the level manipulation for the rhythm deviants, namely a very noticeable shortening of the preceding note, that pose an additional challenge for the selection of a suitable baseline period for the rhythm deviants.

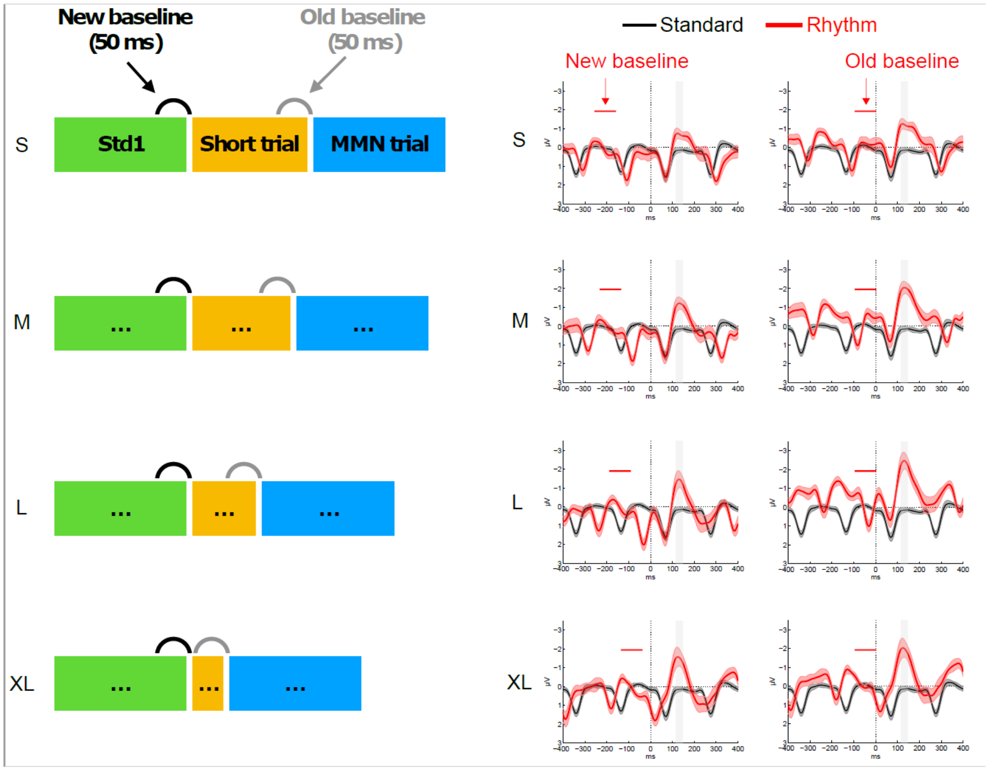

Supplementary Figure 4. Baseline correction for the rhythm deviant waveforms.

5 Timbre deviants and root-mean squared differences in partial tones

The plots below show the root-mean squared (RMS) differences of the first 30 partial tones (for the pitch E with F0 = 330 Hz) of the timbre deviants compared to the standard tone. Also, a CI hearing simulation example is provided, where the sound samples were preprocessed with AngelSim: Cochlear Implant and Hearing Loss Simulator, V1.08.01 (Emily Shannon Fu Foundation) (using the default speech processor settings for 8 CI electrodes), prior to the spectral envelope comparisons. Simulation results suggest abnormal high energy in the spectral envelope, especially between 1-3 kHz, for the bright piano, S, deviants.

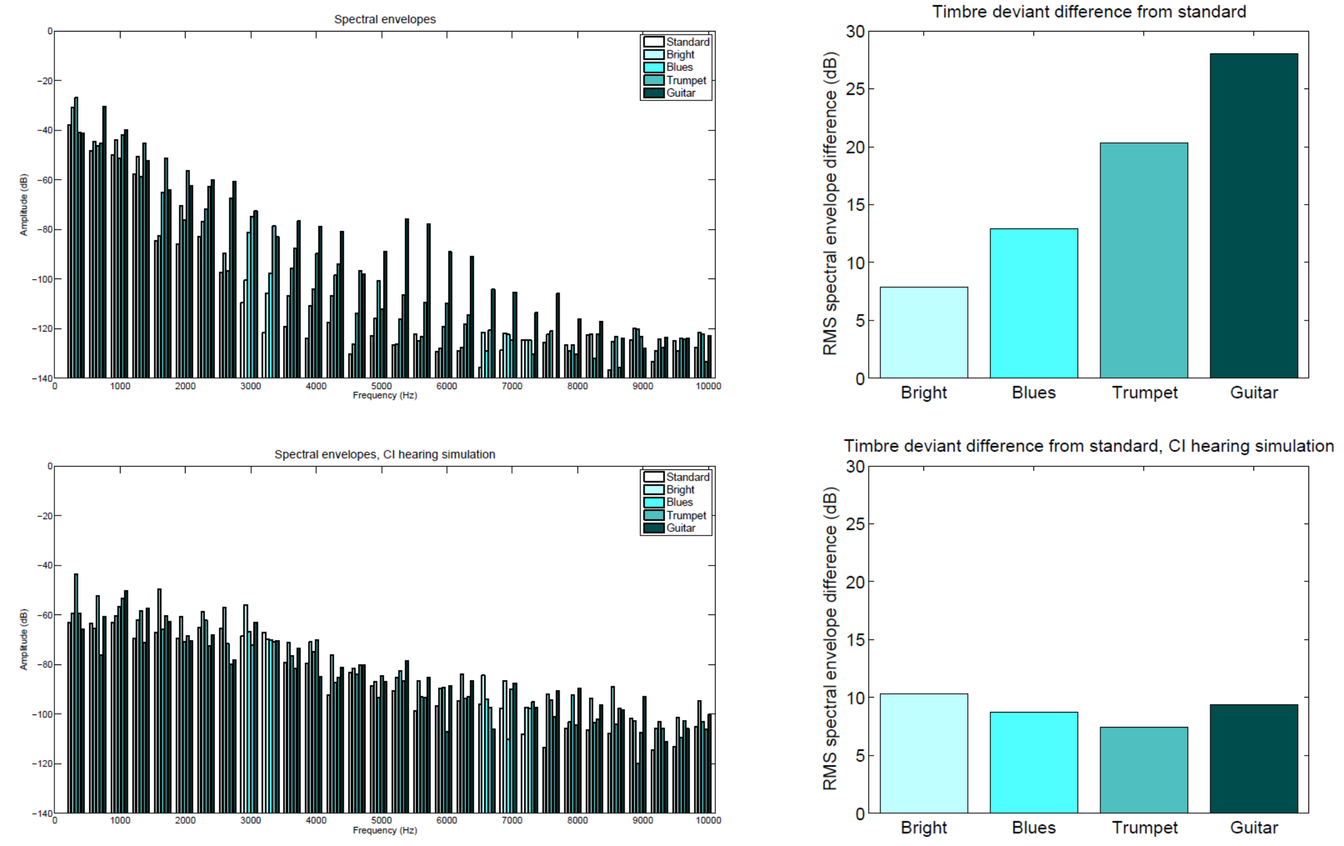

Supplementary Figure 5. Spectral envelopes differences between standard and deviant timbre. Showing the root-mean-squared (RMS) spectral envelope difference in dB between the timbre deviants and timbre standard stimuli. Left: normal results; right: results after preprocessing the sound waveforms with simulation of hearing with a CI.
